# Pulsed Electromagnetic Fields Induce Skeletal Muscle Cell Repair by Sustaining the Expression of Proteins Involved in the Response to Cellular Damage and Oxidative Stress

**DOI:** 10.3390/ijms242316631

**Published:** 2023-11-23

**Authors:** Silvia Maiullari, Antonella Cicirelli, Angela Picerno, Francesca Giannuzzi, Loreto Gesualdo, Angela Notarnicola, Fabio Sallustio, Biagio Moretti

**Affiliations:** 1 Department of Interdisciplinary Medicine, University of Bari “Aldo Moro”, 70124 Bari, Italy; silvia.maiullari@uniba.it (S.M.); antonella.cicirelli@uniba.it (A.C.); angela.picerno@uniba.it (A.P.); francesca.giannuzzi@uniba.it (F.G.); 2 Nephrology, Dialysis and Transplantation Unit, Department of Precision and Regenerative Medicine and Ionian Area (DIMEPRE-J), University of Bari, Piazza G. Cesare 11, 70124 Bari, Italy; loreto.gesualdo@uniba.it; 3 Orthopaedic and Trauma Unit, Department of Translational Biomedicine and Neuroscience “DiBraiN”, University of Bari “Aldo Moro”, Piazza G. Cesare 11, 70124 Bari, Italy; angela.notarnicola@uniba.it (A.N.); biagio.moretti@uniba.it (B.M.)

**Keywords:** PEMF, skeletal muscle cells, cellular damage

## Abstract

Pulsed electromagnetic fields (PEMF) are employed as a non-invasive medicinal therapy, especially in the orthopedic field to stimulate bone regeneration. However, the effect of PEMF on skeletal muscle cells (SkMC) has been understudied. Here, we studied the potentiality of 1.5 mT PEMF to stimulate early regeneration of human SkMC. We showed that human SkMC stimulated with 1.5 mT PEMF for four hours repeated for two days can stimulate cell proliferation without inducing cell apoptosis or significant impairment of the metabolic activity. Interestingly, when we simulated physical damage of the muscle tissue by a scratch, we found that the same PEMF treatment can speed up the regenerative process, inducing a more complete cell migration to close the scratch and wound healing. Moreover, we investigated the molecular pattern induced by PEMF among 26 stress-related cell proteins. We found that the expression of 10 proteins increased after two consecutive days of PEMF stimulation for 4 h, and most of them were involved in response processes to oxidative stress. Among these proteins, we found that heat shock protein 70 (HSP70), which can promote muscle recovery, inhibits apoptosis and decreases inflammation in skeletal muscle, together with thioredoxin, paraoxonase, and superoxide dismutase (SOD2), which can also promote skeletal muscle regeneration following injury. Altogether, these data support the possibility of using PEMF to increase SkMC regeneration and, for the first time, suggest a possible molecular mechanism, which consists of sustaining the expression of antioxidant enzymes to control the important inflammatory and oxidative process occurring following muscle damage.

## 1. Introduction

Skeletal muscle has a sophisticated architecture optimized for efficient function. Its contractile capacity is orchestrated by well-organized myofibers, which originate from the fusion of myoblasts, ultimately forming multinucleated myotubes [1]

Skeletal muscle tissue is renowned for its remarkable regenerative capacity in response to minor injuries or damage. This regenerative capacity is primarily supported by muscle stem cells, known as satellite cells, which lie in a quiescent state but are ready to spring into action to repair and regenerate damaged muscle tissue [2,3]. This self-repair capacity allows skeletal muscle tissue to rapidly recover from trauma and adapt to a variety of external stimuli, maintaining its functionality and structural integrity. However, in the case of more serious injuries or pathological conditions, this regenerative capacity can be compromised, leading to impaired muscle function and a reduced quality of life for those affected. In such circumstances, the identification of effective and non-invasive therapies becomes crucial to promote optimal regeneration and restore muscle function. To this aim, several types of biophysical stimuli have been studied. Mostly, mechanical stimulation [4,5] electrical stimulation [6,7], and their synergistic effect have been explored [8]. While electrical stimulation and the administration of chemical compounds have been widely used in tissue regeneration studies, both approaches present significant limitations. These limitations have prompted scientists to consider alternative approaches. In this context, pulsed electromagnetic fields (PEMF) are emerging as a potential therapeutic option to stimulate regeneration.

In clinical settings, extremely low frequency PEMF are employed as a non-invasive therapy for repair in the orthopedic field. The Food and Drug Administration (FDA) first allowed the use of PEMF in human medicine in 1979 to treat non-union fractures [9].

Osteoporosis fractures and delayed unions are two examples of bone disorders that can be treated with PEMF therapy [10]. By stimulating osteoblastogenesis and influencing calcium storage and mineral metabolism, PEMF increases both bone volume and density [11]. Electromagnetic field applications in rats, human red blood cells, and human hemoglobin cell-free models have shown that these stimuli can increase tissue oxygenation, microcirculation, and angiogenesis [12,13,14]. Possible mechanisms for these reactions include PEMF-induced changes in Ca^2+^/NO/cGMP/PKG signaling [15,16] and the PEMF-induced regulation of nitric oxide signaling [17]. The paracrine role of mesenchymal stem cells is also enhanced by PEMF in cartilage regeneration [18]. Although PEMF have been used for a long time in bone and connective tissue, their effect on skeletal muscle cells (SkMC) has been understudied. PEMF has been shown to increase the myotube diameter and fusion index, activating TRPC1-mediated calcium entry and mitochondrial respiration downstream of calcineurin-dependent transcriptional regulation and P300/CBP-associated factor (PCAF)-epigenetic signaling, which was previously found to be involved in myogenesis [19,20]. Even less is known about the ability of PEMF to accelerate human SkMC regeneration after injury or the chemical mechanism behind this effect [20,21,22]. Here, we studied the potentiality of PEMF to stimulate the early regeneration of human SkMC. PEMF stimulation at a 1.5 mT intensity and a frequency of 75 Hz has been shown to be effective in the activation of various cellular processes. Specifically, recent studies reported significant enhancements in cellular proliferation, differentiation, anti-inflammatory activity, and regeneration in response to 1.5 mT PEMF [23,24,25,26].

Our research aimed to clarify the impact of 1.5 mT PEMF on SkMC previously employed in human tendon cells [27]. We found that PEMF treatment is able to quicken the in vitro repair of SkMC damage by increasing their proliferation and by increasing the production of proteins involved in the response to cellular damage and oxidative stress such as heat shock protein 70, superoxide dismutase 2, thioredoxin-1, and paraoxonases.

## 2. Results

### 2.1. PEMF Exposure Does Not Alter the Metabolic Activity and Viability of Skeletal Muscle Cells

**Figure 1:**
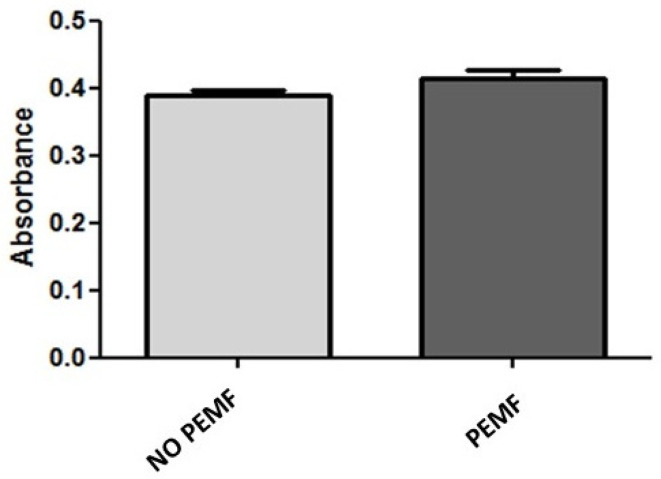
Effect of pulsed electromagnetic fields (PEMF) on the metabolic activity of human skeletal muscle cells (SkMC). MTT assays showed that there was no decrease in cellular metabolic rate after exposure to PEMF for 4 h for two consecutive days. Values are expressed as absorbance mean ± SEM (*n* = 3).

**Figure 2:**
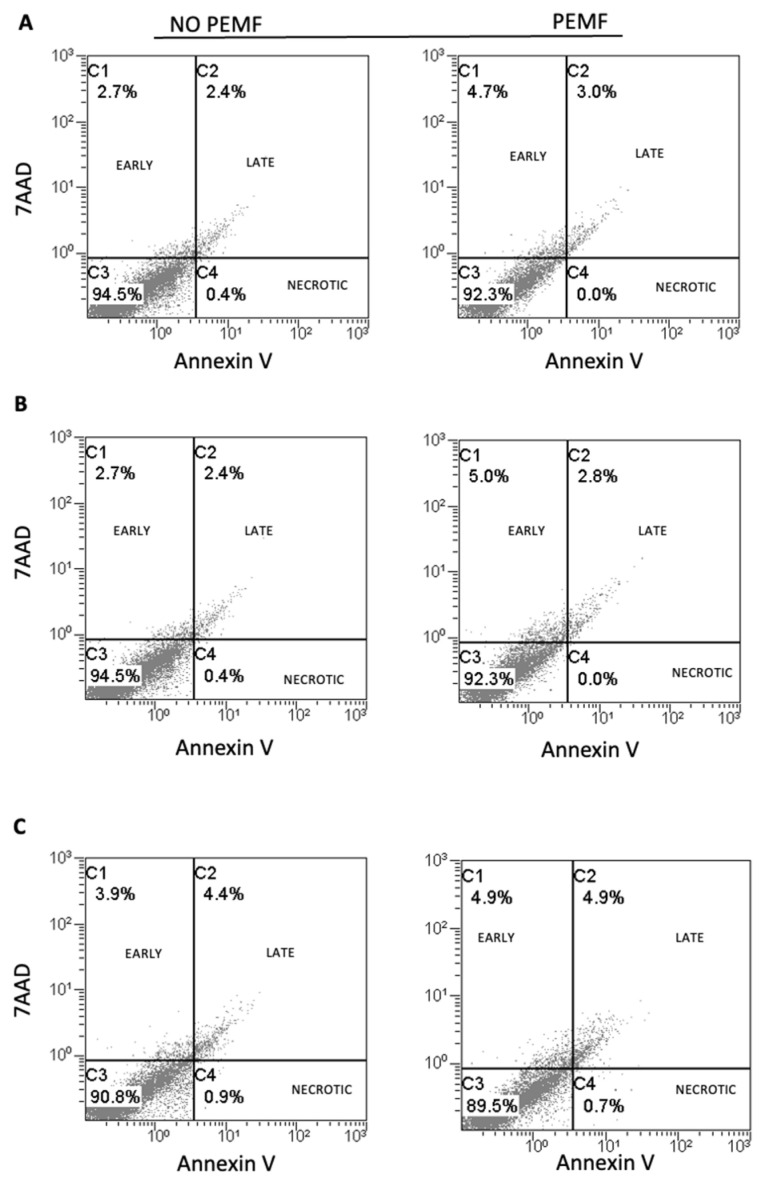
Effect of pulsed electromagnetic fields (PEMF) exposure on cell apoptosis in human SkMC. (**A**) Levels of Annexin V and 7AAD staining determined by FACS after exposure to PEMF for 6 h (**A**), 24 h (**B**), and 48 h (**C**). No significant differences were observed in early apoptosis, late apoptosis, or necrosis between cells treated and untreated with PEMF. Viable cells are AnnexinV^−^/7AAD^−^. The early apoptotic cells are AnnexinV^+^/7AAD^−^. Late apoptosis cells are AnnexinV^+^/7AAD^+^. Data are representative of 3 independent experiments.

We studied whether 1.5 mT PEMF affected the viability of SkMC and performed metabolic activity and apoptosis assays. Thiazolyl blue tetrazolium bromide (MTT assays) showed that the exposure to PEMF for 24 h did not affect the metabolic activity of SkMC. Indeed, the metabolic activity of SkMC exposed to PEMF was the same as that of the not-exposed cells (Figure 1). Since we aimed to evaluate whether PEMF exposure also affects the cells’ viability, we then performed Annexin V-FITC and 7-AAD double staining at 6, 24, and 48 h after PEMF treatment to determine the induced apoptosis (Figure 2A, Figure 2B, and Figure 2C, respectively). We found no significant increase in apoptosis in any of the three-times-treatment groups. In particular, after 48 h of PEMF treatment, the percentage of double negative (Annexin V and 7-AAD cells negative) live cells was 90.8% compared to 89.5% of cells not treated with PEMF (Figure 2C). These data suggest that exposure to PEMF does not increase the apoptosis or necrosis of SkMC.

### 2.2. PEMF Exposure Stimulates Muscle Cells Proliferation

We studied whether PEMF exposure induced an increase in the SkMC proliferation rate. We quantified the proliferation rate of exposed and unexposed cells by measuring the incorporation of BrdU (5-bromo-2′-deoxyuridine, a thymidine analogue) during DNA synthesis in replicating cells.

**Figure 3:**
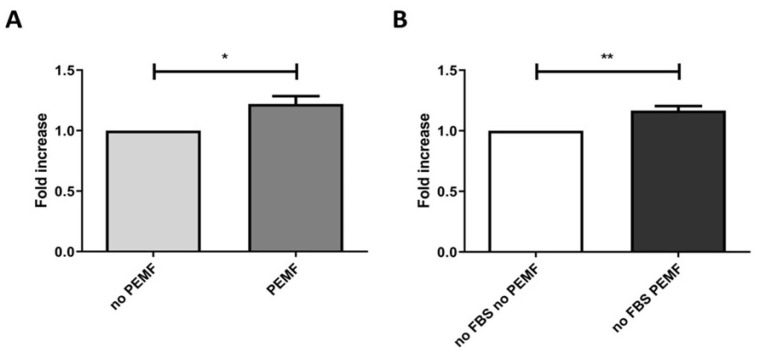
Effect of pulsed electromagnetic fields (PEMF) on skeletal muscle cells (SkMC) proliferation determined by BrdU assay. Muscle cells were treated in magnetic field for 4 h on two consecutive days with or without serum-free culture medium (PEMF—no FBS PEMF). SkMC exposed to PEMF exhibited significantly increased proliferation compared to the control cells under both complete medium conditions (**A**) and serum-free conditions (**B**). The data were normalized to the proliferation of the control cells (no PEMF; no FBS and no PEMF). Data are presented as the mean ± SEM from three independent experiments (* *p* < 0.05, ** *p* < 0.01).

The BrdU assay results suggested that PEMF treatment enhanced muscle cell proliferation by approximately 20% both in cells grown in complete medium (Figure 3A) and in serum-starved cells (Figure 3B), compared to the respective untreated controls.

### 2.3. Exposure to PEMF Results in Increased MyoD Expression

**Figure 4:**
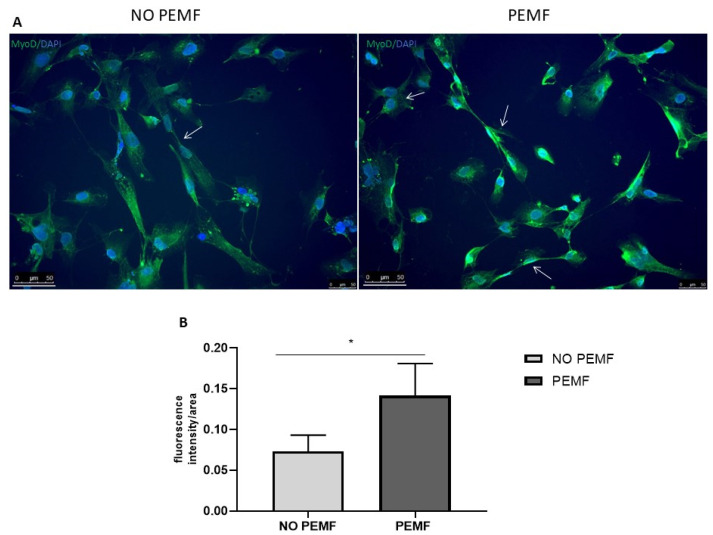
Effect of pulsed electromagnetic fields (PEMF) on MyoD expression in human skeletal muscle cells (SkMC) by immunofluorescence staining. Exposure to PEMF increases MyoD levels. (**A**) Representative immunofluorescence microscopy images of MyoD. Cell nuclei were stained using DAPI. White arrows indicate SkMC fusing to form multi-nucleated myotubes. Scale bars represent 50 µm. (**B**) Quantification of MyoD fluorescence intensity compared to control cells. The intensity was normalized to the immunofluorescence area. Data are presented as the mean ± SEM. The significance of the results was tested using a Student’s *t*-test between groups with a minimum of 6 images (* *p* < 0.05).

Skeletal muscle cell differentiation and regeneration is orchestrated by a network of transcription factors, among which MyoD plays a pivotal role. Therefore, we examined the effects of PEMF stimulation on MyoD expression. Immunofluorescence image analysis revealed a significant increase in MyoD intensity after cell stimulation with PEMF for four hours daily for two consecutive days, compared to untreated controls. Images also showed SkMC fusing to form multi-nucleated myotubes (Figure 4). These results suggest the potential role of PEMF in the induction of muscle differentiation and the intricate balance between myoblast proliferation and differentiation.

### 2.4. Muscle Cells Increase Their Regeneration following Wound Damage When Treated with PEMF

**Figure 5:**
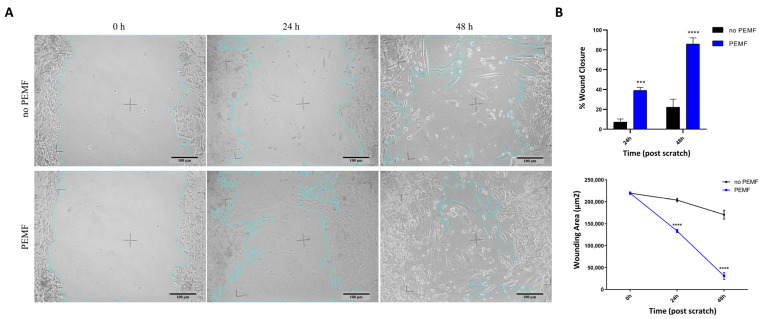
Effect of PEMF exposure on the migratory capacity of immortalized human skeletal muscle cells tested by the scratch assay. PEMF exposure enhances the migratory capacity of skeletal muscle cells, facilitating efficient wound closure. (**A**) Representative images of the scratch areas quantified by the Wound Healing Size Tool, showing the original wound (0 h) and the wound after 24 h or 48 h in cells exposed or not exposed to PEMF. The wound area is marked with a blue line. Scale bar: 100 µm. (**B**) Quantitative analysis of percentage of wound closure (top diagram) and of wounding area (µm^2^) (bottom diagram) at different time points. Data are presented as the mean ± SEM (*n* = 3, *** *p* < 0.001, **** *p* < 0.0001 vs. no PEMF).

To investigate the PEMF capacity to improve SkMC regeneration following damage, wound healing assays were performed. The results showed that the cell repair ability significantly increased after cell stimulation with pulsed electromagnetic fields for 4 h a day for two consecutive days. In fact, the wound area was reduced by approximately 32% (*p* < 0.001) and 64% (*p* < 0.0001) 24 and 48 h after the scratch, respectively, in comparison with PEMF-unexposed controls (Figure 5). These results suggest that PEMF increase the mobility of human skeletal muscle cells, increasing their regenerative capacity following physical damage.

### 2.5. Exposure to PEMF Correlates with Increased Expression of Proteins Involved in Cellular Stress

**Figure 6:**
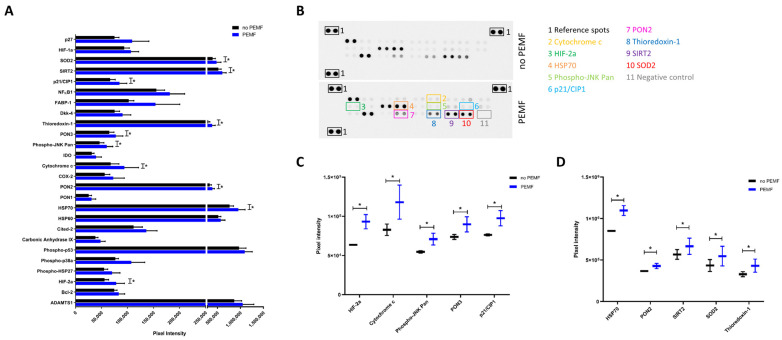
Effect of PEMF exposure on the proteomic profile of cellular stress proteins in human SkMC. (**A**) Graph–bar plot showing the expression of 26 human proteins associated with cellular stress in human SkMC after 4 h of PEMF exposure (blue bars) for two consecutive days compared to no treatment (black bars). Protein signals were quantified by subtracting the signal from the negative control spots (background signal) from each signal of protein spots. Bars indicate mean ± SEM *(n* = 3 independent experiments); * *p* < 0.05 vs. control cells. (**B**) Representative membrane images of the Human Cell Stress Array showing the spots of the proteins in human muscle cells not exposed (top) or exposed (bottom) to PEMF. Color boxes highlighted spots of proteins significantly modulated by PEMF. (**C**) Box plots showing proteins significantly increased by PEMF and expressed at lower level in human SkMC. (**D**) Box plots showing proteins significantly modulated by PEMF and expressed at higher level in human SkMC. Protein signals were quantified by subtracting the signal from the negative control spots (background signal) from each signal of protein spots. Data are presented as the mean ± SEM of three independent experiments; * *p* < 0.05 vs. control cells.

To assess whether exposure to PEMF caused a change in the proteomic profile of cellular stress proteins, we analyzed 26 cell-stress-related proteins. Protein extracts of SkMC treated or untreated with PEMF for 4 h for two consecutive days were analyzed on a proteome array. We identified 10 proteins involved in the response to oxidative stress and the modulation of cell proliferation and apoptosis, which were significantly overexpressed in PEMF-treated cells compared to the controls (Figure 6A,B). In particular, we could distinguish five proteins expressed at a lower level, Phospho-JNK Pan (30% increase over controls), Paraoxonase 2 (PON2) (+18% vs. controls), Sirtuin 2 (SIRT2) (+17% vs. controls), superoxide dismutase 2 (SOD2) (+22 vs. controls), thioredoxin-1 (+28% vs. controls) (Figure 6C), and five proteins expressed at a higher expression level, heat shock protein 70 (HSP70) (+25% vs. controls), hypoxia-inducible transcription factor 2a (HIF-2a) (+40% vs. controls), Cytochrome c (+39% vs. controls), Paraoxonase 3 (PON3) (+19% vs. controls), and p21/CIP1 (+27% vs. controls) (Figure 6D).

**Figure 7:**
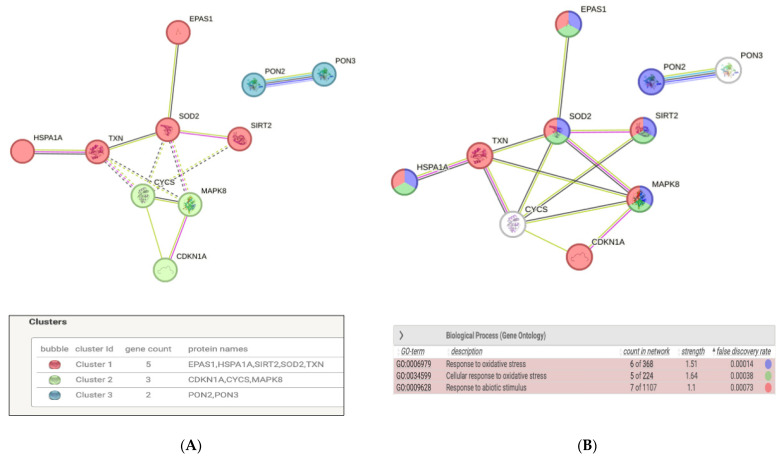
Clustering functional analysis of the 10 proteins modulated by PEMF in SkMC. (**A**) Modulated proteins can form three principal clusters, highlighted with bubbles in red, green, and blue colors. Cluster 1 and 2 are highly interconnected with each other by physical binding or functional interactions. The legend shows the proteins belonging to the same cluster. (**B**) Gene ontology functional enrichment analysis shows that the modulated proteins were enriched in “response to oxidative stress” (proteins denoted with blue color), “cellular response to oxidative stress” (proteins denoted with green color), and “response to abiotic stimulus biological processes” (proteins denoted with red color). The legend shows the significant biological processes enriched by proteins and the corresponding false discovery rate. HSPA1A is HSP70, EPAS1 is HIF-2-Alpha, CDKN1A is p21, and MAPK8 is JNK (aliases).

When we performed functional analysis, we found that proteins modulated by PEMF were interconnected with each other and could form three clusters, two related to the oxidative stress response and one to the cell cycle (Figure 7A). The gene ontology (GO) enrichment analysis showed that the most significant biological processes in which these proteins were involved were “response to oxidative stress” (false discovery rate = 0.00014), “cellular response to oxidative stress” (false discovery rate = 0.00038), and “response to abiotic stimulus” (false discovery rate = 0.00073, Figure 7B and Supplementary Table S1).

## 3. Discussion

Bone health is enhanced by PEMF because of their ability to trigger osteoblastogenesis as well as to influence calcium storage and mineral metabolism [28]. Rats, human erythrocytes, and cell-free tests have all indicated that PEMF can increase tissue oxygenation, microcirculation, and angiogenesis [12,29]. A change in nitric oxide signaling may underlie these reactions [30]. Multiple studies have shown that PEMF can restore homeostatic cell functions like viability, proliferation, differentiation, communication with neighboring cells, and interaction with components of the extracellular matrix by modulating both cell surface receptor expression/activation and downstream signal transduction pathways [31,32,33,34].

However, research into the effectiveness of PEMF stimulation for skeletal muscle tissue therapy is lacking. Only a small number of articles have described the effects of PEMF stimulation on patients’ skeletal muscle [35,36]. PEMF were found to be beneficial in mitigating the physiological deficits linked to the exercise 24, 48, and 72 h following delayed onset muscle soreness (DOMS) induction exercise. These deficits included the improved recovery of perceived muscle soreness, median frequency (MDF), and electromechanical delay (EMD) during isometric contraction. Nevertheless, how PEMF affects DOMS physiologically or clinically has not been studied [35]. Moreover, patients stimulated with PEMF with a local point stimulation were significantly more effective than the non-stimulation group at the fatigue recovery of the quadriceps after regular knee extension/flexion exercise following evaluation through the EMG analysis [36]. However, also in this study, the physiological or molecular mechanisms were not investigated.

Recently, it has been shown that myotube diameter is increased after PEMF stimulation, and adult myosin heavy chain isoform expression is induced in the myotubes [20].

The choice of the intensity of 1.5 mT for pulsed electromagnetic fields (PEMF) stimulation is particularly significant in light of the results observed in other studies. It was, in fact, observed that this specific intensity was actually able to stimulate anabolic processes and limit the catabolic effects of IL-1β in chondrocytes [37], to improve the proliferation and expression of tissue-specific markers in osteoblasts [38,39], and to improve fiber organization, with a decrease in cell density, vascularity, and fat deposition [25]. Finally, stimulation with PEMF at an intensity of 1.5 mT promotes the differentiation and proliferation of myoblasts, through the activation of TRP channels and the influx of calcium into the cells, highlighting their crucial role in facilitating myogenesis [19].

In this study, we showed that human SkMC stimulated with 1.5 mT PEMF for four hours and repeated for two days can stimulate cell proliferation without inducing cell apoptosis or significant impairment of the metabolic activity. Interestingly, when we simulated physical damage of the muscle tissue through a scratch, we found that the same PEMF treatment can speed up the regenerative process by promoting a more complete cell migration to close the scratch and accelerate wound healing. Similar results were reported by Sakhrani et al. using a monolayer of bovine fibroblast-like synoviocytes. Compared to untreated controls, a gradual improvement in cell migration was observed in fibroblast-like synoviocytes treated with PEMF stimulation, with an increase in wound closure percentage at 24 h and 48 h following injury [40].

Furthermore, an increase in MyoD expression was observed following PEMF stimulation. This presents an intriguing link to the differentiation process of skeletal muscle cells. MyoD, a crucial member of the myogenic regulatory factor (MRF) family, plays a critical role in early myogenic differentiation by orchestrating the transcriptional program required for muscle cell commitment and subsequent maturation. Indeed, MyoD is instrumental in the activation of muscle-specific genes, such as the myosin heavy chain (MyHC) and myogenin, thus promoting the transition of myoblasts into myocytes and their subsequent fusion into multinucleated myotubes [41,42,43]. The upregulation of MyoD expression in response to PEMF stimulation suggests its potential involvement in driving the early stages of myogenic commitment and differentiation, thus influencing the cell fate of muscle precursor cells.

This increase in MyoD expression following PEMF exposure aligns with findings from prior studies [19].

Moreover, we investigated the molecular pattern induced by PEMF among 26 cell-stress-related proteins. We found that the expression of 10 proteins increased after two consecutive days of PEMF stimulation for 4 h, and most of them were involved in response processes to oxidative stress. As many as seven of these proteins fell into the “cellular response to abiotic stimulus” gene ontology category (Figure 6B), describing a process that results in a change in the state or activity of a cell (in terms of movement, secretion, enzyme production, gene expression, etc.) as a result of an abiotic (non-living) stimulus. Even if some of the protein-expression-level increase was not very strong following PEMF stimulation, it may still be important when considering all modulated proteins together. Indeed, all these proteins play a role in fighting the oxidative and inflammatory effect produced by chemical or physical damage to muscle cells.

The induction of the protein pattern with anti-oxidative/anti-inflammatory activity is particularly relevant in the muscle repair process. The immune system’s job after an injury is to strike a fine balance between inflammatory and reparative immune cells. Anti-inflammatory cytokines and lipid mediators are crucial to the effective resolution of acute inflammation, which occurs after the immune activation has been triggered in the first stage of damage [44,45]. Persistent tissue damage and an inability to restore homeostasis may result in chronic inflammation, if inflammatory triggers persist, and alter homeostatic set points [45].

We found that one of the most significant PEMF-increased proteins was heat shock protein 70 (HSP70 and HSPA1A). HSP70 can protect cells against damage caused by high temperatures or oxidative stress. Proteins are particularly vulnerable to damage from these stressors, which can lead to their partial unraveling and even aggregation. HSP70 prevents these partially denatured proteins from aggregating and hinders their refolding by transiently attaching to hydrophobic regions exposed to stress [46]. In addition, HSP70 can inhibit apoptosis [47] and has an impact on energy metabolism [48].

In skeletal muscle, HSP70 is required for the normal size and function of muscle fibers, for mantaining Ca^2+^ homeostasis, and for muscle regeneration [49]. HSP70 levels increase during exercise and muscle recovery; conversely, they decrease due to muscle disuse and atrophy [50,51]. Some studies have tried to upregulate HSP70 expression, and this has been shown to protect the muscle against damage, atrophy, and muscular dystrophy and preserve skeletal muscle plasticity and regenerative capacity in aging [49,52].

These beneficial effects of HSP70 could be explained by the role of this stress protein in modulating inflammation in skeletal muscle [49,53].

In particular, Senf et al. demonstrated that HSP70 plays a crucial role in regulating the early inflammatory response to injury in a mouse model. In fact, its expression was essential for the recruitment of immune cells, the phagocytosis of necrotic cell debris, and promoting efficient muscle healing [49].

Additionally, it should be noted that HSP70 functions as a regulator of myogenic differentiation during the early stage of mouse myoblast differentiation, during which an increase in HSP70 protein expression is observed [54,55].

In support of this evidence, a correlation was observed between the knockdown of HSP70 and reduced expression of myogenin, a muscle-specific transcription factor that initiates myogenesis and myoblast differentiation [55,56].

Our results are in line with a previous study showing that, in a rat model of a muscle crush injury, PEMF induced muscle recovery via increased HSP70 protein [53]. To our knowledge, we reveal, for the first time, that PEMF could also play a role in enhancing the expression of HSP70 in human immortalized skeletal muscle cells. The use of PEMF may be beneficial in stimulating the production of this chaperone protein in muscle after injury or during aging, thereby speeding up and improving muscle regeneration.

HSP70 is directly linked with thioredoxin (TXN) and manganese superoxide dismutase (SOD2), which we also found augmented by PEMF. TXN has a radical scavenging activity and protects cells from damage caused by oxidative stress in many different contexts [57]. It contributes to the response to intracellular nitric oxide by inducing the nitrosylation of the active site, the Cys of CASP3, thereby inhibiting caspase-3 activity to protect against oxidative stress [57,58]. Notably, TXN overexpression reduces age-related muscle wasting by preventing oxidative stress and apoptosis in mitochondria [59]. Moreover, TXN was shown to promote hypoxia-induced proliferation of pulmonary artery smooth muscle cells by influencing HIF activation and, in turn, PI3K-Akt activation [60]. Interestingly, in our study, we also found an upregulation of hypoxic factor HIF-2A. In addition, SOD2 was found upregulated after PEMF exposure in SkMC, and it is also induced by TXN [61]. Interestingly, SOD2 overexpression preserves myoblast mitochondrial mass and function in aging mice [62] and can promote skeletal muscle regeneration following injury [63]. This was also demonstrated by the pharmacological stimulation of SOD2, which caused the downregulation of p57, an inhibitor of cyclin-dependent kinase [64]. Paraoxonase 2 (PON2) and 3 (PON3) were also increased by PEMF stimulation in SkMC. Paraoxonases are a family of antioxidant enzymes with anti-inflammatory properties [65], and PON2 not only has an enzymatic function but also is involved in cellular signaling in multiple ways: as a regulator of epithelial Na+ channels [66], an activator of the PI3K/Akt/GSK-3 RISK pathway [67], and an aider of GLUT1-mediated glucose transport [68].

Our findings are in line with previously reported results by the Varani group on different kinds of cells: PEMF were shown to exert a strong anti-inflammatory effect in human chondrocytes and synoviocytes [23,69] as well as anti-oxidative stress activity in neuronal cells through the expression control of another transcription factor driven by hypoxia, HIF-1α, and the inhibition of ROS production [70].

In general, after muscle damage, antioxidant enzymes and reactive oxygen species (ROS) both have an impact on muscle progenitor cell proliferation, differentiation, and maturity. Moreover, skeletal muscle fibrosis is thought to be modulated in part by antioxidant enzymes.

Our data, therefore, support the possibility of using PEMF to enhance SkMC regeneration and, for the first time, suggest a possible molecular mechanism by which PEMF may promote the expression of antioxidant enzymes including TXN, SOD2, and paraoxonase, along with the upregulation of HSP70.

The present study, while providing valuable insights into the effects of PEMF exposure on skeletal muscle cells, had some limitations. First, it is essential to acknowledge that this investigation solely focused on a specific immortalized human skeletal muscle cell line. Therefore, the findings may not fully represent the responses of different skeletal muscle cells, which can exhibit distinct characteristics under various stimuli, and the complexity of in vivo responses. Furthermore, this study primarily examined the effects of PEMF exposure at a specific duration and frequency, neglecting the potential impact of varying exposure times and frequencies. Consequently, the comprehensive effects of diverse PEMF parameters on skeletal muscle cell behavior remain to be fully elucidated. Future investigations encompassing diverse cell types, exposure parameters, and experimental models are necessary to provide a comprehensive understanding of the effects of PEMF on skeletal muscle cells and to further validate the potential clinical utility of PEMF in skeletal muscle tissue regeneration.

## 4. Materials and Methods

### 4.1. Stimulation with Pulsed Electromagnetic Fields (PEMF)

Human skeletal muscle cells were purchased from Applied Biological Materials Inc. (Cat.T0034) and cultured in Skeletal Muscle Cell Growth Medium (Cat.no C-23260; PromoCell, Heidelberg, Germany) supplemented with Skeletal Muscle Cell Growth Medium SupplementMix (Cat.no C-39365; PromoCell, Heidelberg, Germany) and 1% Penicillin–Streptomycin (Cat.no ECB3001; Euroclone, Milan, Italy) at 37 °C with 5% carbon dioxide. These cells were positive for myoblast determination protein 1 (MyoD) and Integrin Alpha-7 (ITGA7) markers. Control tests for cell morphology, adherence rate, and cell viability were performed for each lot of human skeletal muscle cells by the vendors. Immunohistochemical tests for cell-type-specific markers were also carried out for each lot.

For the stimulation of cells with PEMF, we used a device provided by IGEA suitable to stimulate cells in culture condition. Two copper wire Helmholtz coils placed on opposing sides of a plexiglass container were used to build the generators. Then, 6-well plates or 25 cm flasks containing cells were placed in the plexiglass container in the cell incubator, and PEMF were applied for 4 h. Then, after 20 h, PEMF were applied again for four hours, and the cells were left to stand for further 20 h before proceeding with the experiments. Within the culture region, a consistent magnetic field was produced using a signal generator. The PEMF device was calibrated to clinical standards using a magnetic field peak strength of 1.5 ± 0.2 mT, a pulse duration of 1.3 ms, and a frequency of 75 Hz.

All PEMF-stimulated cells were compared with PEMF-unexposed control samples, that were manipulated in the same experimental conditions.

### 4.2. MTT Assays

The metabolic activity of SkMC in normal conditions and after exposition to pulsed electromagnetic fields PEMF was measured by the MTT Cell Proliferation Assay Kit, according to the manufacturer’s instructions (Cat.no CGD1; Sigma, St. Louis, MO, USA). Cells were plated in 6-well plates at 2 × 10^5^ cells/well, and then they were treated or not treated with PEMF for four hours for two consecutive days.

Then, MTT solution (5 mg/mL in phosphate-buffered saline with pH 7.2; Sigma, St. Louis, MO, USA) was added in an amount equal to 10% of the culture volume, and cells were incubated for 2 h at 37 °C. The resulting MTT formazan crystals were dissolved using MTT solvent (with 0.1 N HCl in anhydrous isopropanol) in an amount equal to the original culture volume. The supernatant was then transferred to a 96-well plate, and the optical density (OD) was measured with a microplate reader at 570 nm.

### 4.3. Apoptosis Assays

To evaluate the percentage of early apoptotic, necrotic, and viable cells, SkMC were stained with Annexin V-FITC/7-AAD Kit (Cat.no IM3614; Beckman-Coulter, Brea, CA, USA). The cells were harvested, rinsed twice with 1× PBS, and then suspended in 200 µL of 1× binding buffer, followed by the addition of 10 µL of Annexin V–FITC solution and 20 µL of 7-AAD Viability Dye. After a 15 min incubation on ice in the absence of light, the stained cells were collected, rinsed with PBS, and suspended in the incubation buffer. The cell suspension was evaluated with an FC500 flow cytometer (Beckman-Coulter, Brea, CA, USA) and analyzed using Kaluza analysis software (software version 1.2; Beckman-Coulter, Brea, CA, USA). 

### 4.4. Cell Proliferation Assays

Cell proliferation assays were performed by ELISA BrdU assay (Cat.no 11647229001; Roche Diagnostics GmbH Indianapolis, IN, USA). First, 6 × 10^3^ muscle cells were seeded in four different flat-bottomed 96-well microplates and, after 4 h of PEMF stimulation for two consecutive days, BrdU was added (10 µM) to all wells. After a further incubation of 24 h, the plates were dried at +60 °C for 1 h.

The incorporated BrdU SkMC were fixed and permeabilized using the FixDenat provided in the kit. Anti-BrdU antibody (Anti-BrdU-POD working solution) was added to the wells and incubated for 90 min at room temperature. After three washes, 100 µL of Substrate Solution containing Tetramethylbenzidine (TMB) was pipetted into each well. After approximately 30 min, the absorbance was measured at a wavelength of 450 nm using an ELISA reader for microplates (Multiskan FC Microplate Photometer, cat. no 51119000; Thermo Scientific Inc., Waltham, MA, USA).

### 4.5. Scratch Assays

First, 2 × 10^5^ SkMC/well were seeded in independent 24-well plates and incubated at 37 °C and 5% CO_2_. A scratch in the monolayer (full confluence) was induced with a sterile 10 μL pipette tip, and the complete medium was replaced to eliminate detached cells. We incubated the cells at 37 °C with 5% CO_2_ and stimulated the plates with PEMF for 4 h for two consecutive days.

The images were acquired immediately after and 24 h and 48 h after the scratch (t_0_, t_24_, and t_48_, respectively) under a light microscope with a microscope digital camera (AmScope MU1000 10MP). A marker drawn on the bottom of each well was used to locate the same position captured by the camera.

Image analysis was carried out using the Wound Healing Size Tool plugin [71] in ImageJ 1.54d software (National Institutes of Health, Bethesda, MD, USA; http://rsb.info.nih.gov/ij/). The percentage of wound closure at different time points was calculated according to the equation

$$\mathrm{WoundClosure}\%=\frac{A_{t0}-A_{\Delta t}}{A_{t0}}\times 100\%,$$

where A_t0_ is the initial wound area, and A_Δt_ is the wound area after n hours (24 h or 48 h) of the initial scratch, both in μm^2^ [71].

Each experiment was performed in triplicate.

### 4.6. Immunofluorescence Staining

Cell monolayers with or without PEMF treatment were fixed in 4% PFA in PBS, permeabilized in 0.5% Triton X-100 (Cat.no 9036-19-5; Sigma-Aldrich, St. Louis, MO, USA), and blocked in 5% BSA in PBS. Overnight incubation with rabbit anti-MyoD primary antibody (Cat.no D8G3; Cell Signaling Technology, Beverly, MA, USA) diluted 1:200 was followed by goat anti-rabbit IgG H&L secondary antibody (Alexa Fluor 488) incubation. DAPI was added to the cells before analysis by fluorescence microscopy. Fluorescence intensity was quantified using the corrected total cell fluorescent intensity plugin in ImageJ, and results were normalized to each specific area.

### 4.7. Proteome Profile

Twenty-six cells’ stress-related protein expression was analyzed using Proteome Profiler Human Cell Stress Array (Cat.no Ary018; R&D Systems Minneapolis, MN, USA).

SkMC of three different cell passages were cultured up to 60% confluence at 37 °C and 5% CO_2_ and divided into two groups: untreated cells (control; no PEMF) and cells exposed to PEMF for 4 h for two consecutive days. On the third day, the cells were harvested, counted, and lysed with Lysis Buffer 6, included in the kit and supplemented with 10 μg/mL of Protease Inhibitor Cocktail (Cat.no P8340; Sigma-Aldrich, St. Louis, MO, USA), for 30 min on ice. Protein concentration was determined using the Pierce BCA Protein Assay Kit (Thermo Fisher Scientific). Each array was loaded with 250 μg of cell lysate. The subsequent steps were made according to the manufacturer’s recommendations. Chemiluminescent signals were captured using the ChemiDoc MP Imaging System (Bio-Rad Laboratories, Hercules, CA, USA). Pixel density was evaluated using Image Lab Software (software version 6.0.1; Bio-Rad Laboratories, Hercules, CA, USA). Spot signals were normalized by subtracting the signal from negative spots present in each membrane. After background subtraction, the corresponding signals on treated and control arrays were compared to determine relative protein expression changes in cell-stress-related protein.

### 4.8. Statistical Analysis

To compare the differences between controls and PEMF-exposed cells in MTT, Annexin V-FITC/7-AAD, and BrdU assays, we used paired *t*-test. Data are presented as mean ± SEM (standard error of the mean) of independent experiments or samples (*n* ≥ 3). Sidak’s and Tukey’s multi-group comparison tests were performed by two-way analysis of variance (ANOVA), alpha = 0.05, in wounding area and percentage of wound closure of scratch assay analysis, respectively. Data are presented as mean ± SEM of *n* = 3 independent experiments; *p* values are expressed as **** *p* < 0.0001. To analyze the results of the proteome profiler array we used multiple paired t-tests to perform multiple comparisons. *p* values are expressed as * *p* < 0.05, ** *p* < 0.01. Data are presented as mean ± SEM of 3 independent samples (*n* = 3). Statistical analysis and graphs were performed using GraphPad Prism software 11 (GraphPad Software, San Diego, CA, USA).

Clustering and GO functional enrichment analysis of modulated proteins were performed by STRING software (https://www.string-db.org, accessed on 6 August 2023), a database of known and predicted protein–protein interactions, including direct (physical) and indirect (functional) associations. This database contains information from numerous sources, including experimental repositories, computational prediction methods, and public text collections. Each interaction is associated with a combined confidence score that integrates the various pieces of evidence. The Markov Cluster Algorithm was used for the clustering.

## Data Availability

Data are contained within the article in Section 2 and Supplementary Materials.

## Supplementary Materials

The supporting information can be downloaded at https://www.mdpi.com/article/10.3390/ijms242316631/s1.
