# Supplementary material for: Pulsed Electromagnetic Fields Induce Skeletal Muscle Cell Repair by Sustaining the Expression of Proteins Involved in the Response to Cellular Damage and Oxidative Stress
Source: Int J Mol Sci. 2023 Nov 23;24(23):16631. doi: 10.3390/ijms242316631 (PMC10706358; doi:10.3390/ijms242316631)
Supplement: Supplementary file 1 [file ijms-24-16631-s001.zip › ijms-2689709-supplementary.pdf]

Supplementary Table S1. Gene ontology (GO) enrichment analysis showing the most significant biological processes in which PEMF modulated proteins were involved.

| #term ID | term description | observed ger | background g | strength | false discove | matching proteins in the nnetwork |
|---|---|---|---|---|---|---|
| GO:0006979 | Response to oxidative stress | 6 | 368 | 1.51 | 0.00014 | SIRT2,EPAS1,HSPA1A,MAPK8,SOD2,PON2 |
| GO:0034599 | Cellular response to oxidative stress | 5 | 224 | 1.64 | 0.00038 | SIRT2,EPAS1,HSPA1A,MAPK8,SOD2 |
| GO:0009628 | Response to abiotic stimulus | 7 | 1107 | 1.1 | 0.00073 | SIRT2,EPAS1,TXN,HSPA1A,MAPK8,CDKN1A,SOD2 |
| GO:2000377 | Regulation of reactive oxygen species metabolic process | 4 | 143 | 1.74 | 0.0019 | SIRT2,PON3,CDKN1A,SOD2 |
| GO:0043618 | Regulation of transcription from RNA polymerase II promoter in response to stress | 3 | 49 | 2.08 | 0.0054 | SIRT2,EPAS1,HSPA1A |
| GO:0009636 | Response to toxic substance | 4 | 229 | 1.54 | 0.0074 | PON3,TXN,SOD2,PON2 |
| GO:0051173 | Positive regulation of nitrogen compound metabolic process | 8 | 3166 | 0.7 | 0.0259 | SIRT2,EPAS1,CYCS,TXN,HSPA1A,MAPK8,CDKN1A,SOD2 |
| GO:0051098 | Regulation of binding | 4 | 375 | 1.32 | 0.0404 | SIRT2,TXN,MAPK8,CDKN1A |
| GO:0051101 | Regulation of DNA binding | 3 | 124 | 1.68 | 0.0404 | SIRT2,TXN,MAPK8 |
| GO:0051247 | Positive regulation of protein metabolic process | 6 | 1512 | 0.89 | 0.0404 | SIRT2,CYCS,TXN,HSPA1A,MAPK8,CDKN1A |
| GO:0097201 | Negative regulation of transcription from RNA polymerase II promoter in response to stress | 2 | 14 | 2.45 | 0.0404 | SIRT2,HSPA1A |
| GO:0031399 | Regulation of protein modification process | 6 | 1560 | 0.88 | 0.0443 | SIRT2,EPAS1,TXN,HSPA1A,MAPK8,CDKN1A |
| GO:0033554 | Cellular response to stress | 6 | 1572 | 0.88 | 0.0443 | SIRT2,EPAS1,HSPA1A,MAPK8,CDKN1A,SOD2 |
| GO:0044093 | Positive regulation of molecular function | 6 | 1587 | 0.87 | 0.0443 | SIRT2,CYCS,TXN,HSPA1A,MAPK8,CDKN1A |
| GO:0007005 | Mitochondrion organization | 4 | 445 | 1.25 | 0.0458 | EPAS1,HSPA1A,MAPK8,SOD2 |
| GO:0009314 | Response to radiation | 4 | 444 | 1.25 | 0.0458 | TXN,MAPK8,CDKN1A,SOD2 |
| GO:0010941 | Regulation of cell death | 6 | 1651 | 0.85 | 0.0458 | SIRT2,TXN,HSPA1A,MAPK8,CDKN1A,SOD2 |
| GO:0031667 | Response to nutrient levels | 4 | 461 | 1.23 | 0.0458 | SIRT2,MAPK8,CDKN1A,SOD2 |
| GO:0051246 | Regulation of protein metabolic process | 7 | 2622 | 0.72 | 0.0458 | SIRT2,EPAS1,CYCS,TXN,HSPA1A,MAPK8,CDKN1A |
| GO:0070887 | Cellular response to chemical stimulus | 7 | 2609 | 0.72 | 0.0458 | SIRT2,EPAS1,PON3,TXN,HSPA1A,MAPK8,SOD2 |
| GO:0097193 | Intrinsic apoptotic signaling pathway | 3 | 166 | 1.55 | 0.0465 | CYCS,CDKN1A,SOD2 |
| GO:1904706 | Negative regulation of vascular associated smooth muscle cell proliferation | 2 | 25 | 2.2 | 0.0487 | CDKN1A,SOD2 |
